# In vivo observation of Langerhans cells by laser confocal microscopy in Thygeson's superficial punctate keratitis

**Published:** 2009-07-29

**Authors:** Koji Kawamoto, Tai-ichiro Chikama, Norihisa Takahashi, Teruo Nishida

**Affiliations:** Department of Ophthalmology, Yamaguchi University Graduate School of Medicine, Yamaguchi, Japan

## Abstract

**Purpose:**

To characterize the cornea of individuals with Thygeson’s superficial punctate keratitis (TSPK) at the cellular level by laser confocal biomicroscopy.

**Methods:**

Both corneas of three patients with TSPK referred to Yamaguchi University Hospital were imaged with a laser confocal biomicroscope. Morphological changes were evaluated for each layer of the cornea.

**Results:**

The number of Langerhans cells was greatly increased in the basal cell layer of the focal corneal epithelium and in Bowman’s layer in the four eyes affected by TSPK. Aggregates of these cells were associated with the subepithelial nerve plexus. Langerhans cells were also evident in the unaffected eyes of the two patients with unilateral TSPK, although their numbers were much smaller than those in the affected eyes. Topical treatment with betamethasone phosphate resulted in the virtual disappearance of Langerhans cells from the affected eyes.

**Conclusion:**

The prominent association of Langerhans cells with TSPK suggests that the activation of these cells by inflammatory conditions might contribute to the pathogenesis of this disorder.

## Introduction

Thygeson’s superficial punctate keratitis (TSPK) is characterized clinically by ocular irritation, tearing, photophobia, and visual disturbance but has minimal clinical manifestations in the cornea [1-3]. It usually develops bilaterally, and slitlamp microscopic examination reveals aggregates of punctate fluorescein staining in the corneal epithelium that correspond to elevated and coarse granular opacities [2]. Although the etiology of TSPK remains unclear, viral infection, allergic reactions, and immune responses to viral infection have been proposed to play a pathogenesis role. Lubricant eyedrops, eyedrops containing a low dose of corticosteroid, and soft contact lenses are options for medical management. In vivo imaging of eyes affected by TSPK has been performed by confocal microscopy [4]. The images revealed highly refractive microdots and refractive bodies immediately below the corneal epithelium, in Bowman’s layer, and in the anterior stroma. Morphological changes of keratocytes were also apparent in the previous reports.

The Heidelberg Retinal Tomograph II, Rostock Cornea Module (HRTII-RCM; Heidelberg Engineering, Heidelberg, Germany) has recently been introduced into clinical practice in ophthalmology [5-9]. Given that its light source is a laser, the HRTII-RCM has provided images of individual cells of the cornea with high resolution. It has thus revealed the presence of Langerhans cells (LCs), a type of antigen-presenting cell, in the cornea. These cells are located in the peripheral and, to a lesser extent, central regions of the healthy cornea. The number of LCs in the central cornea increases, however, in association with ocular inflammation or after photorefractive keratotomy [10,11]. Both immature and mature LCs have been detected in the healthy corneal epithelium and tend to predominate in the peripheral and central regions, respectively [12]. Whereas immature LCs are not able to present antigen to lymphocytes, their stimulation under inflammatory conditions renders them competent in this regard and thereby promotes immune responses.

We described the characterization of three patients with TSPK by in vivo imaging with the HRTII-RCM in this present report. Our findings suggest that immunological reactions mediated by LCs may contribute to the pathogenesis of TSPK.

## Methods

### Patients

**Figure 1:**
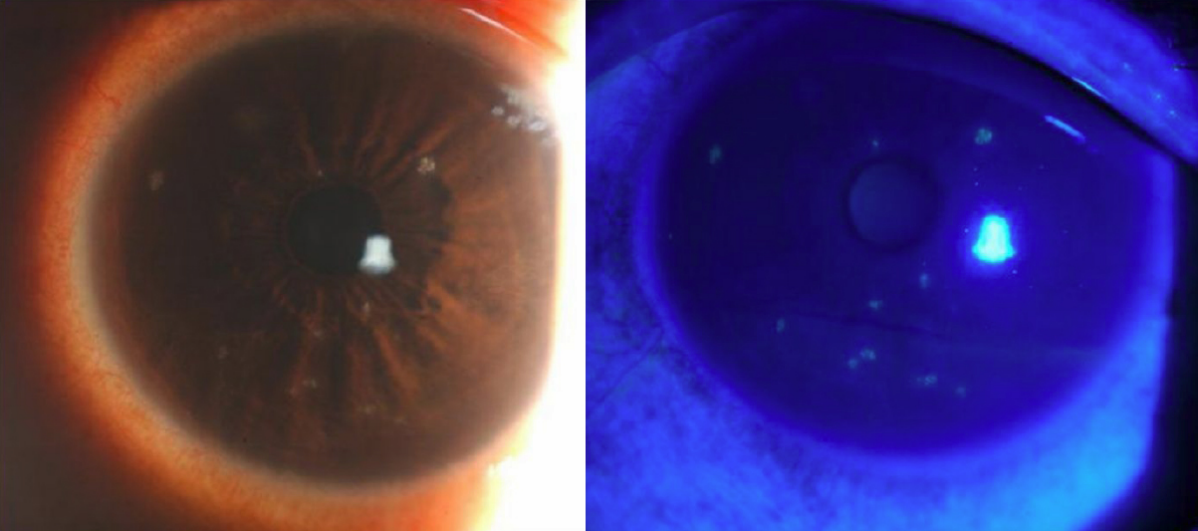
Representative photographs of the affected eyes with TSPK. In the left eye of patient #1, multiple coarse granular opacities stained with fluorescein are seen in the cornea.

Three unrelated patients with TSPK referred to the cornea service at Yamaguchi University Hospital were enrolled in the study. They reported ocular irritation, tearing, photophobia, or foreign-body sensation in one (two patients) or both (one patient) eyes. Slitlamp microscopic examination revealed that characteristic elevated and coarse granular opacities that stained with fluorescein in the affected eyes (Figure 1). Corrected visual acuity of the affected eyes was 30/20 in two instances and 20/20 in one instance. Photokeratoscope images revealed irregularity of the corneal surface at the sites of the opacities. On the basis of these clinical manifestations, we diagnosed the three patients with TSPK. Treatment with topical betamethasone phosphate ophthalmic solution (four times a day for 4 weeks) ameliorated the subjective symptoms and other clinical manifestations of each patient. The characteristics of the patients are summarized in Table1.

**Table 1:** Clinical characteristics of patients with TSPK.

**Patient**	**Age (years)**	**Sex**	**Subjective symptoms**	**Clinical manifestation (slitlamp examination)**	**Corrected visual acuity**	**Treatment**
#1	31	M	Bilateral irritation, tearing, photophobia	Aggregation of elevated coarse granular opacities (fluorescein staining positive)	OD: 30/20 OS: 30/20	Topical betamethasone phosphate
#2	49	F	Unilateral (OS) foreign-body sensation	Grayish elevated coarse granular opacities (fluorescein staining positive)	OS: 30/20	Topical betamethasone phosphate
#3	45	F	Unilateral (OD) irritation, tearing, photophobia	Grayish elevated coarse granular opacities (fluorescein staining positive)	OD: 20/20	Topical betamethasone phosphate

### HRTII-RCM imaging

With written informed consent, we examined both corneas of each patient by HRTII-RCM imaging on their first visit to our clinic. The study was also approved by the Institutional Review Board of Yamaguchi University Hospital.

A drop of local anesthetic (0.4% benoxinate hydrochloride; Santen, Osaka, Japan) was instilled into the lower fornix of each eye, and a drop of polymer gel (Viscotears; CIBA Vision, Duluth, GA) was applied to the microscope probe to allow for optical coupling of the microscope objective lens to the cornea. HRTII-RCM imaging was performed at the central and focal cornea including the coarse granular opacities. Images of all layers of the cornea were collected and saved. All images were analyzed with a personal computer (12 inch monitor with a display resolution of 1024 by 768 pixels) and under the same lighting conditions so as to avoid variation in image contrast on the monitor. Three observers viewed the digitally stored images frame by frame in a masked manner. The most representative images from each corneal layer were selected for evaluation and ranked according to the severity of change.

## Results

Most of the abnormalities in the eyes affected by TSPK were confined to the basal cell layer of the corneal epithelium, the subepithelial nerve plexus, Bowman's membrane, and the anterior stroma, with the middle and deep regions of the stroma and endothelial cells being largely normal. The changes observed were similar in the four affected eyes of the three patients but they varied in severity.

### Corneal epithelium

**Figure 2:**
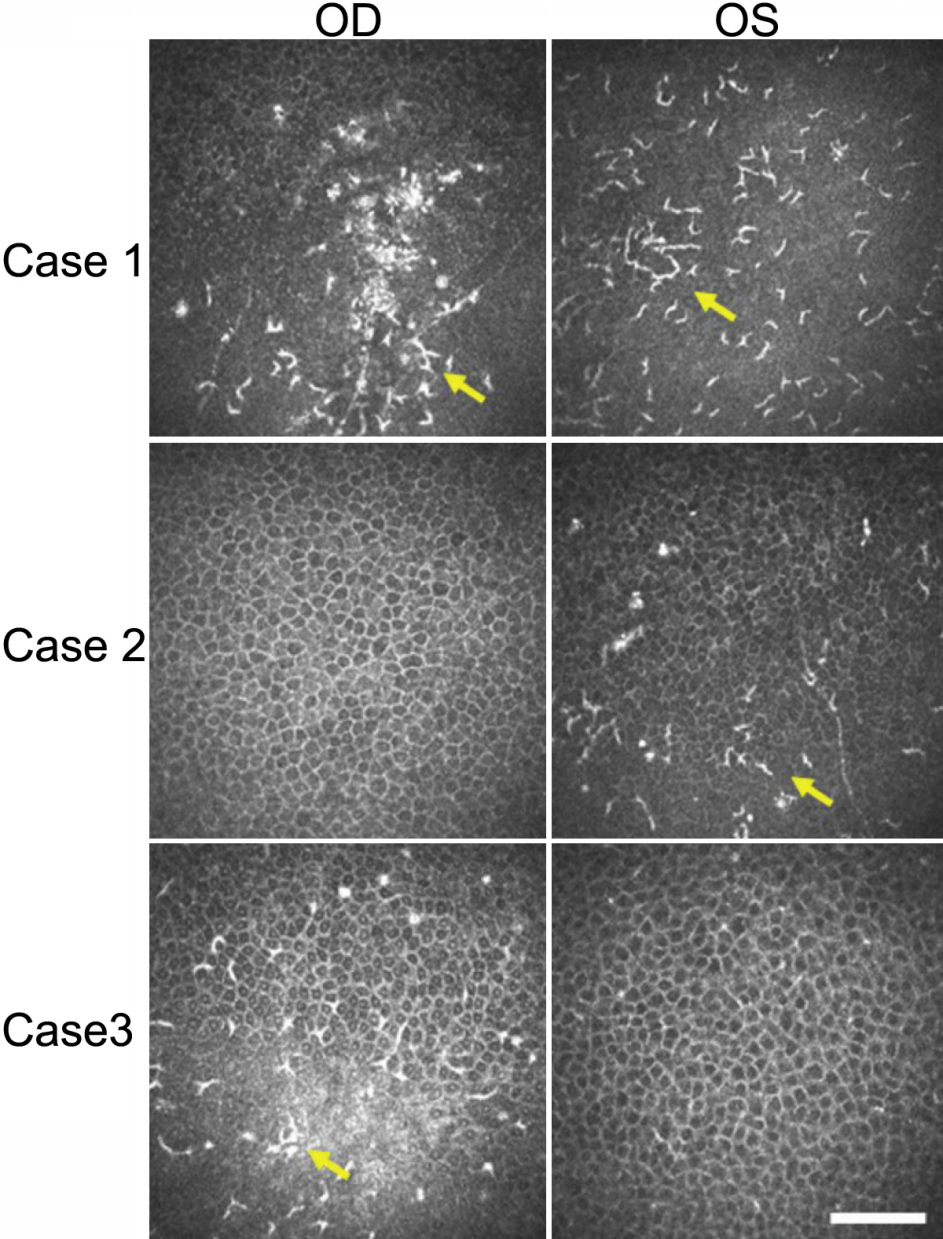
Representative HRTII-RCM images of the basal cell layer of the corneal epithelium in three patients with TSPK. All images show the honeycomb-like appearance characteristic of the normal cornea. Highly refractive dots and aggregation of dendritic cells (arrow) were also evident in both eyes of patient #1, in the left eye (OS) of patient #2, and in the right eye (OD) of patient #3. Scale bar, 50 μm.

Superficial cells of the corneal epithelium showed no substantial changes in cell size, shape, or brightness (data not shown) in the local region including the coarse granular opacities of the affected eyes. In wing cell layer and basal cell layer, we could not detect any abnormalities in cell shape and cell number. In contrast, an increase in the number of dendritic cells was apparent in the basal cell layer of the corneal epithelium in all affected eyes (Figure 2). The number of such dendritic cells differed among the three patients in a manner apparently independent of age, gender, or pattern of disease onset. The number of highly refractive cell bodies was also increased in the affected corneas in all cases. Small numbers of dendritic cells or highly refractive cell bodies were also apparent in the basal cell layer of the corneal epithelium of the unaffected eyes of patients #2 and #3. The average LC density of affected and unaffected eyes by TSPK is 232±109 cell/mm^2^ and 15.0±2.21 cell/mm^2^ in the basal cell layer, respectively. The number of LC in TSPK affected eyes significantly increased.

### Bowman’s layer

**Figure 3:**
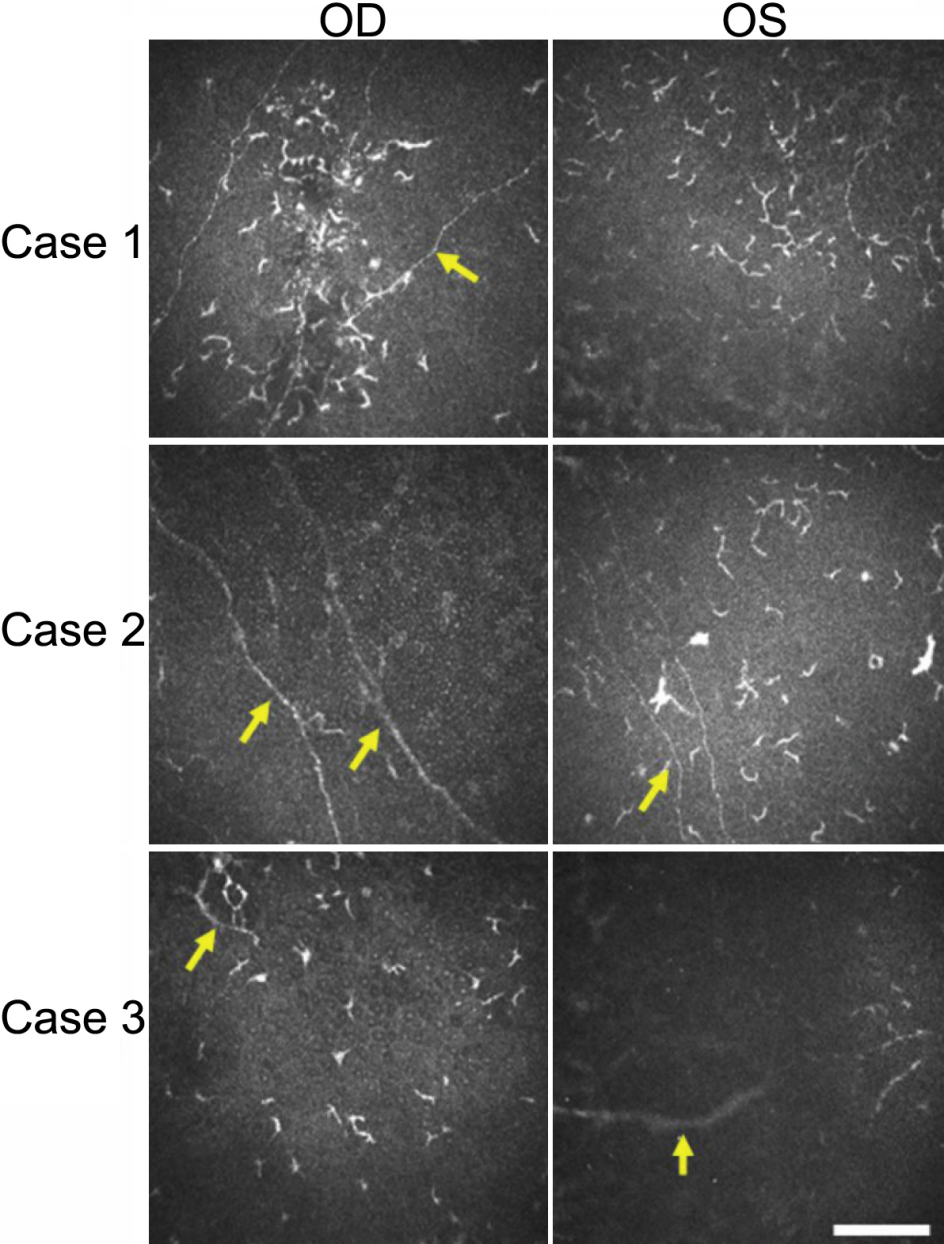
Representative HRTII-RCM images of the subepithelial nerve plexus of the cornea in three patients with TSPK. Aggregated dendritic cells were apparent in association with the nerve plexus (arrow) in both eyes of patient #1, in the left eye of patient #2, and in the right eye of patient #3. Scale bar, 50 μm.

Dendritic cells were detected in association with the subepithelial nerve plexus immediately above Bowman's membrane in the eyes affected by TSPK (Figure 3). These cells aggregated in parallel to the nerve fibers in both affected eyes of patient #1 and #2, but such arrangement was faintly apparent in patient of #3. Several dendritic cells were also observed in the unaffected eyes of patients #2 and #3. The average LC density of affected and unaffected eyes by TSPK 160±20.6 cell/mm^2^ and 21.9±4.42 cell/mm^2^ in the nerve plexus, respectively. Variability in the morphology of dendritic cells was not obvious in the unaffected eyes of these two patients (Figure 3). Aggregates of mature LCs were detected in association with subepithelial nerve fibers in the central cornea of affected eyes.

### Corneal stroma and endothelial cells

Some keratocytes had highly reflective cell bodies of irregular size, orientation, and shape in the anterior stroma in affected eyes (data not shown). These abnormalities were most pronounced in the region closest to Bowman's membrane. Images of the posterior stroma and corneal endothelium appeared normal in the affected eyes.

### Post-therapeutic images

**Figure 4:**
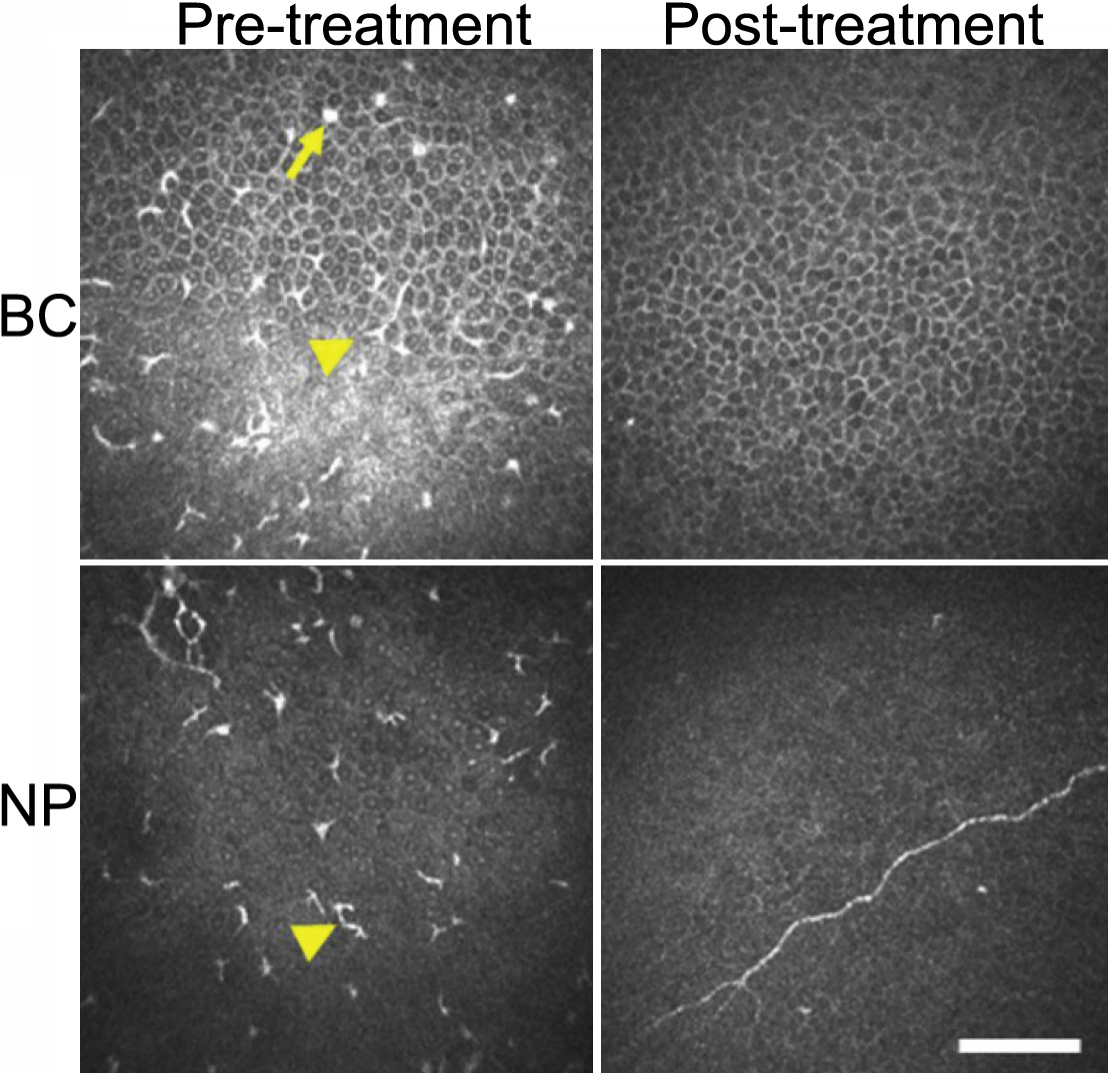
Representative HRTII-RCM images of the right cornea of patient #3 before and after treatment. The dotlike opacities (arrow) and dendritic cells (arrow head) apparent in the basal cell layer of the corneal epithelium (BC) or in association with the subepithelial nerve plexus (NP) before treatment were no longer evident after treatment. Scale bar, 50 µm.

We also obtained post-therapeutic HRTII-RCM images for patient #3 (Figure 4). The numbers of dendritic cells in the basal cell layer of the corneal epithelium and in Bowman’s membrane of the affected eye were greatly reduced after treatment. The overall appearance of these cell layers was also now similar to that in the unaffected eye in the same patient. No dendritic cells were detected in association with the subepithelial nerve plexus immediately above Bowman's membrane in the treated eye.

## Discussion

With the use of HRTII-RCM, we have obtained in vivo images of the central cornea in eyes affected by TSPK. Although dendritic cells were observed in the unaffected healthy corneas of the two patients with unilateral TSPK, these cells were greatly increased in number and formed aggregates in both the basal cell layer of the corneal epithelium and in Bowman’s layer of the affected eyes. The dendritic cells in Bowman’s layer appeared associated with the nerve fibers that form the nerve plexus of the corneal epithelium. Medical intervention reduced the numbers of these dendritic cells to normal values.

With use of HRTII-RCM, we were able to observe abnormalities of cell shape and cell density in the eyes affected by TSPK. In the affected eyes, accumulation of dendritic cells was apparent in the focal lesions. Tandem scanning confocal microscopy of eyes affected by TSPK previously revealed multiple highly reflective filamentous structures in Bowman’s layer of the corneal epithelium, but these structures were not conclusively identified as dendritic cells [4]. In addition, no inflammatory cells were detected in the stroma in this previous report. In contrast to in vivo tandem scanning confocal microscopy, the HRTII-RCM is able to provide high-resolution images of each layer of the cornea. With this instrument, we were thus able to demonstrate the accumulation of dendritic cells in the subepithelial, Bowman’s lesions, and anterior stroma. Dendritic cells have been shown by tandem scanning confocal microscopy to invade the basal cell layer of the corneal epithelium in eyes infected with herpes simplex virus [13,14]. LCs have been observed by the HRTII-RCM both in the healthy cornea and in the cornea of eyes affected by viral infection or contact lens wearing [11,12]. By comparison with the characteristic images of LCs, we conclude that the dendritic cells detected in the affected eyes of our patients with TSPK were LCs, most likely mature LCs, which are capable of a rapid response to pathogens such as viruses or to injury of the corneal epithelium or stroma.

Electron microscopy has previously revealed dendritic cells in close contact with nerve fiber bundles in the cornea of individuals with ocular viral infection [15]. In the skin, LCs are modulated by neuropeptides released from epidermal nerves [16,17]. The aggregation of LCs in association with the subepithelial nerve plexus in the cornea of TSPK patients detected in the present study suggests that these cells might also be regulated by neuropeptides released from the nerve fibers. The disappearance of the LCs after treatment with an anti-inflammatory ophthalmic solution is consistent with the notion that TSPK is an immunological disease.

In conclusion, we have demonstrated the accumulation and aggregation of LCs in the basal cell layer of the corneal epithelium and in association with the subepithelial nerve plexus in the affected eyes of patients with TSPK. These observations suggest that TSPK might result, at least in part, from immune responses to an unknown inciting agent.
